# The Forever War: understanding, science fiction, and thought experiments

**DOI:** 10.1007/s11229-019-02306-6

**Published:** 2019-07-15

**Authors:** Harald A. Wiltsche

**Affiliations:** Department for Culture and Communication, Linköping University, 581 83 Linköping, Sweden

**Keywords:** Thought experiments, Science fiction, Understanding

## Abstract

The aim of this paper is to show that scientific thought experiments and works of science fiction are highly suitable tools for facilitating and increasing understanding of science. After comparing one of Einstein’s most famous thought experiments with the science fiction novel “The Forever War”, I shall argue that both proceed similarly in making some of the more outlandish consequences of special relativity theory intelligible. However, as I will also point out, understanding in thought experiments and understanding in science fiction differ in one important respect: While the former aim at what I shall call “physical understanding”, science fiction novels typically have “existential understanding” as their target.

## Introduction

Looking back at the past three decades, the philosophical debate about thought experiments (“TEs”) is characterized by a strong focus on their alleged knowledge-producing powers (cf., for an overview, Brown and Fehige 2017). This might seem surprising, especially in light of the historical record. Although it is certainly correct that some TEs were intended to facilitate knowledge about reality, more often than not TEs in science seem to have served other purposes, such as disclosing inconsistencies in existing theories or, perhaps even more important, making consequences of theories easier to understand. Given this, and given the strong focus on knowledge production in the existing literature in philosophy of science, it might be worth paying closer attention to how understanding of theories and/or the phenomena in their domain is facilitated through TEs.

As has been recently noted (Stuart 2018), such a shift in attention gains additional plausibility from recent work in mainstream philosophy of science and epistemology. The epistemic value of understanding has been marginalized for the better part of the twentieth century. To some extent, this was due to the general fixation with propositional knowledge that characterizes much of Western philosophy. In philosophy of science, the neglect of understanding was further exacerbated by the assumption that understanding is merely a subjective byproduct of explanation, a feature that was taken to be describable in terms of objective relations between theory and evidence alone.^1^ In recent years, however, the philosophical winds have shifted. Nowadays a growing number of philosophers argue that understanding is an epistemic good that needs to be studied in its own right or, even more forcefully, that understanding ought to supplant knowledge and explanation as the focus of philosophical attention (e.g. Kvanvig 2003). Though space does not permit me to enter this discussion here (cf., e.g., De Regt et al. 2005; De Regt 2017; Grimm et al. 2017), it should be apparent that the “recovery” of understanding in mainstream philosophy also has consequences for the discussion about scientific TEs: If understanding is not just a feeling, but one of the principal epistemic aims of science, and if, furthermore, TEs are particularly apt to increase understanding, then coming to terms with the reasons for this aptness is without doubt an important task (cf. also Stuart 2016).

There is yet another reason to look more closely at the ways in which TEs facilitate understanding. Recent years have seen increasing attention to TEs, but also to the relations between TEs and other areas of philosophical interest. For instance, following early suggestions by Davenport (1983) and Sorensen (1992), philosophers have started to explore the connections between TEs and works of literary fiction (cf., e.g., Carroll 2002; Davies 2007; Swirski 2007; Elgin 2007, 2014; De Smedt and De Cruz 2015; Egan 2016). The presumption that such a comparison might be fruitful is indeed a natural one: Like TEs, works of literary fiction require their readers to cognitively participate in fictional narratives in which hypothetical or counterfactual scenarios are described. And like TEs, some works of literary fiction allow us to learn something about reality, even though they are usually written without “fidelity constraint” (Davies 2007, p. 31), i.e. without the requirement to include only events that are believed to have occurred. However, in this discussion too, a strong emphasis on knowledge production does not seem to be the most natural starting point. Of course, there is a trivial sense in which we do acquire propositional knowledge about reality from works of literary fiction: Since it contains much factual information about life in the middle ages, a lot about medieval Italy can be learned from reading Eco’s *The Name of the Rose*. And whenever we have finished reading a work of fiction, we have thereby gained knowledge that we have just read a work of fiction. However, such trivialities aside, it strikes me as odd to say that the cognitive outcome of my reading of *Nineteen Eighty-Four* is an increase in my stock of true propositions about the physical world. Even if I did not learn anything new about the world, Orwell’s novel may help me to *understand* how the corruption of the media aids totalitarian regimes.^2^

With these generalities in mind, let me say a word about the aim and the structure of my paper. In part one, I will try to get a grip on the kind of understanding that is brought about in scientific TEs. To this end, I shall discuss Einstein’s train, a TE that is commonly used to elucidate one of the more counter-intuitive consequences of special relativity theory (“SR”). In part two, I will give a brief summary of Joe Haldeman’s science fiction novel *The Forever War* (“TFW”). One of the intriguing aspects of TFW is that it depicts a fictional world in which technological innovations make the effects of SR directly experienceable. Based on the comparison between Einstein’s train and TFW, I shall argue that both proceed similarly in making fairly outlandish consequences of SR intelligible. However, as I will point out in the final part of my paper, understanding in TEs and understanding in science fiction nevertheless differs in one important respect: While TEs in physics aim at what I shall call *physical understanding*, science fiction novels like TFW have *existential understanding* as their target.

## Einstein’s train

Science has a history of challenging deeply entrenched empirical intuitions about reality. Many intuitions that were thought to reflect substantial metaphysical insights turned out to be of limited reliability because they were formed under non-generalizable conditions. For instance, our experiences are usually limited to objects that are moving with velocities much lower than the speed of light. Although intuitions that are formed on this experiential basis may be adequate to account for most everyday phenomena, they fail to provide a conceptual basis for a scientific treatment of motion in general. The revolution in physics that occurred at the beginning of the twentieth century helps to make this point vivid.

Following Einstein’s canonical presentation, SR rests on two explicit postulates. The first, referred to as the *principle of relativity*, is known since the times of Galileo and states that the laws of physics are the same in every inertial frame of reference. The second postulate, referred to as the *light principle*, is a consequence of the first: If Maxwell’s equations hold in all inertial frames, then the only possible value of the speed of light in all inertial frames is *c*. Building on these two postulates, Einstein introduces a definition of simultaneity according to which two events, e_1_ and e_2_, are simultaneous if and only if light flashes emitted from both events meet exactly at the spatial midpoint between e_1_ and e_2_.

Taken in isolation, the two postulates and the definition may appear innocent enough: While the principle of relativity states that the outcome of any given experiment is independent from the inertial frame in which it is performed, the light principle states that the velocity of light is constant in all inertial frames, and independent from the velocity of the emitting source. And once the constancy of *c* is accepted, Einstein’s definition of simultaneity follows naturally. In conjunction, however, the two postulates yield several perplexing consequences: it follows, for instance, that the question whether two spatially distant events occur at the same time cannot be answered in an absolute, frame-independent sense. In the next paragraphs, I shall discuss two strategies to bring this consequence, which is usually referred to as the *relativity of simultaneity*, to the fore. While the first is to derive the relativity of simultaneity from the mathematical formalism of SR, the second relies on one of Einstein’s most celebrated TEs. A comparison between the two strategies will help to gain a clearer grasp of the kind of understanding that is brought about in TEs.

A crucial component of modern physical theories are the transformation rules that describe the relationship between the coordinates (*x*, *y*, *z*, *t*) and ($x^{\prime },y^{\prime },z^{\prime },t^{\prime }$) of a set of events, as measured in two inertial frames *S* and ${S^{\prime }}$ that differ only in their constant relative motion, with velocity *v*. In pre-relativistic physics, the coordinates are related to one another by the Galilean transformations, which are given by the equations

$$\begin{aligned} x^{\prime }=x-vt,\quad y^{\prime }=y,\quad z^{\prime }=z,\quad t^{\prime }=t. \end{aligned}$$

The final equation $t^{\prime }=t$ codifies the pre-relativistic intuition that time is invariant under Galilean transformations and that, consequently, the temporal order of two events e_1_ and e_2_ always stays the same, no matter from which reference frame e_1_ and e_2_ are measured. Note, however, that the Galilean transformations are incompatible with the postulates of SR. If $t^{\prime }=t$ and $x^{\prime }=x-vt$, then parallel velocities add and no velocity can be invariant across inertial frames that are in motion with respect to each other. This means that, contrary to the light principle, the value of *c* would change depending on the relative motion of the inertial frame from which *c* is measured. As a consequence, the Galilean transformations must be replaced by transformation rules that are compatible with the postulates of SR (and with the implicit assumption that space is homogenous and isotropic). These conditions are satisfied by the Lorentz transformations that are given by the equations

$$\begin{aligned} x^{\prime }=\gamma (x-vt),\quad y^{\prime }=y, \quad z^{\prime }=z, \quad t^{\prime }=\gamma \left( t-\frac{vx}{c^2}\right) , \end{aligned}$$

where

$$\begin{aligned} \gamma =\frac{1}{\sqrt{1-\frac{v^2}{c^2}}}. \end{aligned}$$

Upon applying the Lorentz transformations to relate the coordinates of e_1_ and e_2_, one realizes that the question whether e_1_ and e_2_ occur simultaneously cannot be answered in an absolute, frame-independent way. Assume that e_1_ and e_2_ have identical values of the *t*-coordinate for an observer at rest. A little bit of algebra reveals that the same events e_1_ and e_2_ will have different values of the ${t^{\prime }}$-coordinate if measured by an observer in uniform relative motion with respect to the first.

The point of the previous paragraph was to illustrate that one of the more counter-intuitive consequences of SR can be directly read off from its mathematical core. I shall say more on this approach in a moment. But let me first turn to the second strategy, that of demonstrating the relativity of simultaneity through a TE.

**Fig. 1:**
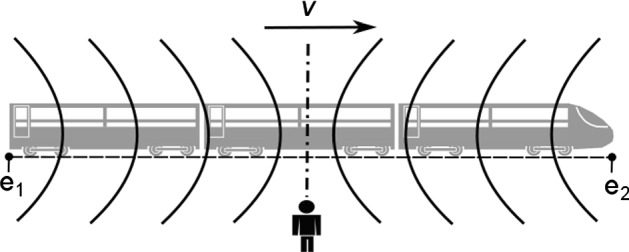
The situation for the second observer

In one of his most celebrated TEs, Einstein (2005, pp. 25–27) asks us to imagine a train that is traveling along a straight track with constant velocity *v*. Let e_1_ and e_2_ be two separate events that emit light flashes at opposite ends of the train. Suppose furthermore that an observer is standing next to the train tracks right at the spatial midpoint between e_1_ and e_2_. Since both light flashes travel with the constant velocity *c*, both arrive at the observer’s position at the exact same time. The observer thus concludes that e_1_ and e_2_ must have happened simultaneously (cf. Fig. 1).

**Fig. 2:**
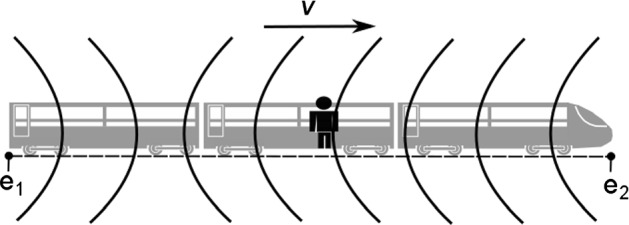
The situation for the second observer

But now imagine a second observer who experiences the same situation from within the train. Although the second observer is also initially located at the spatial midpoint between e_1_ and e_2_, the light from e_1_ arrives at her position a little bit later than the light from e_2_. It is easy to see why (cf. Fig. 2). Light from both sources requires time to reach the observer’s position. But during that time the train moves towards e_2_ with velocity *v*, which means that light from e_1_ has a longer distance to cover until it catches up with the moving observer. Hence, since the light signals from e_1_ and e_2_ arrive at her location at different times, the moving observer rightly judges that e_1_ and e_2_ did not happen simultaneously. Upon reflecting on how the same scenario plays out for two observers in different inertial frames, we thus arrive at the desired conclusion: The simultaneity of two distant events is relative to a frame.

Physics textbooks typically feature discussions of both strategies to demonstrate the relativity of simultaneity. It is interesting to see, however, that in virtually all cases the mathematical derivations are given *after* the basic idea has been introduced through a TE. The reason has to do with the differences between the kinds of understanding that are brought about by the two strategies outlined above. But before I can argue for this claim, some general remarks about the cognitive achievement of understanding are in order.

Broadly speaking, I subscribe to a “manipulationist” account according to which the crucial aspect of understanding is its *empowering role*: Unlike those lacking understanding, understanders are in a position to effectively *use* whatever it is they have understood. This intuition has been elaborated by a number of thinkers, for instance by Hasok Chang, who ties understanding to the performability of an “epistemic activity” (Chang 2009). Following Chang’s usage of the term, an epistemic activity is a “coherent set of mental or physical actions (or operations) that are intended to contribute to the production or improvement of knowledge in a particular way, in accordance with some discernible rules” (Chang 2011, p. 209). On this view, then, a cognitive agent understands *P* (an object, a theory, a model, an equation, a concept, a technique, an instrument, etc.), if she has the skills to perform the epistemic activities that are relevant to *P*. Using the terminology employed by Hills (2016), the intuition underlying Chang’s proposal may also be expressed in terms of “cognitive control” or “cognitive grasp”:^3^ To understand *P* not only means to be in possession of a set of true propositions pertaining to *P*. In order to understand *P*, a cognitive agent must also have the abilities to bring *P* under her cognitive control by *engaging in relevant epistemic activities* with respect to *P*, i.e. by manipulating *P*, by giving an explanation of why *P* or by applying the relevant abilities to ${P^{\prime }}$, where ${P^{\prime }}$ is sufficiently similar to but not identical with *P*.

Perhaps the most sophisticated manipulationist account comes from Daniel Wilkenfeld. Building on a proposal by De Regt and Dieks (2005) and in line with Chang’s views on the matter, Wilkenfeld agrees that understanding ought to “be characterized largely in terms of what it enables one to do” (Wilkenfeld 2013, p. 1000). However, for reasons which will become clear in a moment, Wilkenfeld goes beyond other existing accounts by introducing the additional condition that in order to understand *P*, a cognitive agent must also possess (or be able to construct) a mental representation of *P*. Of course, not every mental representation will do. According to Wilkenfeld, “a mental representation counts as being ‘of the right sort’ in virtue of the fact that possession of it enables one to perform (typically, but not always, intellectual) feats relevant in that context” (Wilkenfeld 2013, p. 1000). Hence, on this view, a cognitive agent understands *P* if, first, she is able to construct a mental representation of *P* and if, second, she is able to perform the epistemic activities that are relevant to (the mental representation of) *P*.^4^

On the view just described, demonstrating the relativity of simultaneity using the Lorentz transformations certainly counts as an epistemic activity in Chang’s sense of the term. In order to obtain the desired result, a cognitive agent must perform certain operations whose execution requires a particular mathematical skillset. Moreover, an assessment of these operations is made on the basis of discernible rules, in this case the rules governing the correct manipulation of mathematical symbols. But does the fact that the cognitive agent performs the right kinds of operations ensure that she succeeds in obtaining understanding? A closer look reveals that this is not the case: Facing the challenge of applying the Lorentz transformations to two quadruples of coordinates, a cognitive agent could behave in all the ways we would expect her to behave. Yet, if all the agent does is to mechanically reproduce a series of pre-memorized operations, then this would hardly count as a display of genuine understanding. Hence, in order to distinguish such cases of “counterfeit understanding” from cases of genuine understanding, cognitive agents are also required to be able to construct a mental representation of whatever it is they seek to understand.^5^ Manipulating this representation allows the agent to produce variations of the initial representation: For instance, the agent could apply the Lorentz transformations to different quadruples of coordinates; or she could use varying representations to detect and correct errors in her derivations. Hence, the upshot of these considerations is that the performability of certain operations is not sufficient for genuine understanding. A cognitive agent must also be able to construct a mental representation to which the relevant operations can be applied.

Let’s assume that a cognitive agent is able to construct a mental representation of the mathematical demonstration of the relativity of simultaneity. And let’s also assume that the agent is able to manipulate this representation by performing the right kinds of mathematical operations. If these two conditions are met (and if the manipulationist account outlined so far is correct^6^), the strategy to derive the relativity of simultaneity from the mathematical formalism of SR yields understanding. Yet, my suggestion is that, given the context in which the relativity of simultaneity is of primary interest, this strategy yields the *wrong kind of understanding*, especially for cognitive agents who are not yet familiar with the specifics of SR. The reason for this verdict is simple: Although you can’t have the former without the latter, physics is more than pure mathematics. Starting from the equations of the Lorentz transformations, a cognitive agent who is sufficiently conversant with mathematics will have no trouble performing the correct operations to obtain the desired results. She can do this, however, without paying attention to the physical meaning of the symbols she is manipulating, or, even more fundamentally, without considering whether the symbols have extra-mathematical meaning at all. But since the point of the whole exercise is not to test or sharpen our mathematical abilities, the kind of *mathematical* understanding that results from manipulating abstract symbols is at best indirectly relevant to the agent’s *physical* understanding of SR.

We may summarize the previous paragraph as follows: Although demonstrating the relativity of simultaneity on purely mathematical grounds yields mathematical understanding, this kind of understanding is not sufficient to promote the physical intelligibility of SR. One of the main reasons is, as I have argued, that a mathematical demonstration leaves the physical meaning of the abstract symbols undetermined. It is this indefiniteness that makes it hard for a cognitive agent to understand the physical significance of the results, even if she possesses the mathematical skills to solve the equations with ease. Of course, one may wonder whether this drawback could be overcome by simply laying down definitions for the symbols and concepts used. Suppose, for instance, that a cognitive agent is able to perform the mathematical operations, but is unfamiliar with the physical concept “reference frame”. Could the cognitive agent gain physical understanding if she were told that a reference frame is defined as a “set of coordinates used by an observer to describe space-time” (Ellis and Williams 1998, p. 27) or as “a coordinate grid equipped with a set of clocks located at the grid intersections and synchronized” (Faraoni 2013, p. 16)? Although it seems too strong of a claim that textbook definitions of this kind are entirely without epistemic significance, the information they provide hardly suffices to render the physical implications of the mathematical derivations intelligible. The reason is that knowing the definition of “reference frame” does not entail the practical knowledge that is necessary to do epistemic things with reference frames, e.g. to map them onto real-world scenarios in order to solve empirical puzzles. This point has been emphasized by thinkers such as Michael Polanyi (1998) or Thomas Kuhn: Since textbook definitions typically do not specify how and under which conditions abstract symbols and concepts can be applied to reality, a student learns their physical meaning “less from the incomplete though sometimes helpful definitions in his text than by observing and participating in the application of these concepts to problem-solution” (Kuhn 1970, p. 47).^7^

As mentioned earlier, it is a common practice in physics textbooks to give a mathematical derivation of the relativity of simultaneity only after the basic idea has been introduced through a TE. We are now in a position to explain why this is so. A mathematical demonstration falls short of providing physical understanding because mathematical operations alone are not suited for consolidating the physical meaning of the abstract symbols and concepts. Yet, it is precisely this consolidation of physical meaning that is accomplished by TEs in a very efficient and undemanding way (cf., on this point, also Stuart 2017, pp. 22–24). Consider again a cognitive agent who is unfamiliar with the concept of a reference frame. While, as we have seen, textbook definitions are only of limited value, performing Einstein’s train TE puts the agent in a position to grasp the physical meaning of the concept in an intuitive, almost playful manner. How does the TE achieve this? In order to perform the TE, the cognitive agent must begin with constructing a mental representation of an imaginary scenario. Like with most other scientific TEs, the scenario is built up from familiar every-day objects such as wagons, train tracks and wave crests that represent propagating light flashes. Once the mental representation of the TE-scenario is set up in the expected manner, the cognitive agent is required to let the scenario unfold, and to record the outcome from two different perspectives. Upon doing this, she not only realizes that the temporal order of the incoming light flashes varies depending on her location within the imaginary scenario. By determining two origins from which the same occurrence is qualitatively described, the cognitive agent also performs the necessary operations to grasp the basic idea of a (non-technical notion of) reference frame.

On the view defended here, TEs facilitate physical understanding by achieving two related objectives. The first is to consolidate the meaning of abstract concepts by requiring the cognitive agent to perform operations through which these concepts are mapped onto mental representations of scenarios that are built up from familiar objects of our every-day experience.^8^ The point of this exercise is to bring unfamiliar concepts under the agent’s cognitive control: The agent comes to understand a new concept by constructing a mental representation of a scenario that she already knows and to which the unfamiliar concept can be successfully applied. If the agent succeeds, she is in a position to do further epistemic things with the newly acquired concept. For instance, by rebuilding the mental representation of the TE-scenario with cars instead of trains, the agent could apply the concept “reference frame” to a scenario that is sufficiently similar but not identical with the original one. Or the cognitive agent could work through a series of imaginary scenarios with decreasing degrees of specificity in order to establish semantic connections between the mathematical formalism and empirical reality.^9^

Once the necessary conceptual resources are in place, it becomes possible to proceed with the second objective. Now that the world is sufficiently well represented conceptually, the cognitive agent can actually perform the TE in order to clarify the consequences that result from applying the newly acquired theoretical framework. There are two reasons why TEs are particularly well suited for this purpose: On the one hand, TEs rely on highly simplified environments for their execution. It is through the omission of uncontrollable factors and outside influences that TEs deliver results with perfect precision and clarity. And this makes it significantly easier to focus on the implications of the theory without being sidetracked by ambiguities with which real experiments are always fraught. On the other hand, TEs make it fairly easy to distort reality in ways that help closing the gap between theoretical concept and empirical fact. It is, for instance, well known that many relativistic effects such as time dilation or length contraction are almost imperceptible at everyday speeds. The train TE makes up for this by stipulating a world in which the succession of incoming light waves is straightforwardly observable without the need for sophisticated measuring devices.

Let me briefly summarize the main results of this section. The process of facilitating physical understanding through TEs involves two key aspects: While constructing a mental representation of the TE-scenario helps fixing the meaning of the relevant theoretical concepts, the actual performance of the TE clarifies the consequences of applying the newly acquired framework. Note, however, that, on the view proposed here, TEs must be actively *done* and not just passively “consumed”: It is, of course, true that the TE-narrative contains most of the information that is relevant for how and in which imaginary world the TE is supposed to be executed. But if understanding crucially depends on the possession of a mental representation of whatever it is one seeks to understand, cognitive agents are required to actively construct the TE-scenarios in their mental spaces.^10^ Let us now, with these remarks as a backdrop, turn to Joe W. Haldeman’s award-winning novel *The Forever War*.

## The Forever War

TFW is a military science fiction novel that was first published in 1974. Its author, Joe W. Haldeman, received a degree in physics and astronomy from the University of Maryland in 1967. In the same year Haldeman was drafted into the US Army and served as a combat engineer in Vietnam. He was severely wounded in battle and received a Purple Heart for his service. TFW is a semi-autobiographical text in which Haldeman reflects on his own wartime experiences as well as on his experiences as a returning veteran (cf. Gordon 2006).

Like Haldeman himself, the main protagonist of TFW, William Mandella, is conscripted by the UNEF (United Nations Exploratory Force) after receiving his degree in physics and astronomy. The year is 1997 and humanity is at war. Yet, the war humanity is fighting in the parallel universe of TFW is quite different from the wars we know. Twelve years earlier, in 1985, scientists had confirmed the hypothesis according to which separate points in spacetime are linked by Einstein–Rosen-bridges or, as they are more familiarly known, “wormholes”. Yet, far from being a purely theoretical success, this discovery had far-reaching practical consequences, for scientists also found a way to enter wormholes through what Haldeman calls “collapsars”:

Just fling an object at a collapsar with sufficient speed, and out it pops in some other part of the galaxy. It didn’t take long to figure out the formula that predicted where it would come out: it travels along the same “line” (actually an Einsteinian geodesic) it would have followed if the collapsar hadn’t been in the way—until it reaches another collapsar field, whereupon it reappears, repelled with the same speed at which it approached the original collapsar. Travel time between collapsars... exactly zero. (Haldeman 1974, p. 7)

With this knowledge in hand, it was possible to cover the vast distances of interstellar space, and humanity could finally colonize the galaxy. But it wasn’t until long that something unexpected happened: a ship with human colonists disappeared and UNEF came to the conclusion that it was destroyed by a hitherto unknown alien race, called the “Taurans”. Humans retaliated and this led to the interstellar conflict into which the main protagonist William Mandella—a somewhat resigned character with pacifist leanings—is thrown.

The fact that humanity is fighting against aliens is not the only reason why the war in TFW is different from the wars we know. For our purposes here, an even more important reason is that the frontline of the conflict can only be reached through collapsar jumps. Now, it is not the jumps themselves that cause problems—as we have seen, spaceships just need to enter a collapsar with sufficient speed and at the right angle in order to re-appear at the other end of the galaxy without any loss of time. The problem is rather to get to the collapsars in the first place. Collapsars are few and far between and in order to cover the vast distances of empty space, spaceships must travel for significant periods of time with velocities close to *c*. However, at such high velocities a peculiar relativistic effect known as *time dilation* becomes manifest. Since this effect plays a crucial role in TFW, and since Haldeman simply stipulates the existence and ramifications of time dilation, it should be discussed in a bit more detail.

The breakdown of absolute simultaneity is but one of several perplexing consequences of SR. Another is *time dilation*, i.e. the phenomenon that a relatively moving observer will measure time differently from an observer at rest. In light of what has been said in the previous section, the best way to make this phenomenon intelligible is by introducing an epistemic practice to fix the meaning of the relevant concepts, and by then working through a TE.

**Fig. 3:**
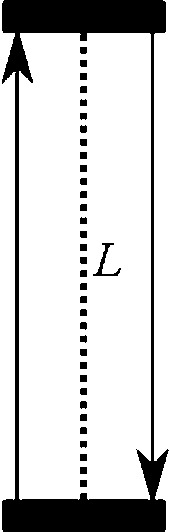
Light clock at rest

Imagine an idealized timekeeping device, a so-called light clock. A light clock consists of a rod with a mirror at each end. A light pulse is traveling back and forth between the mirrors, and every time the light pulse hits the bottom mirror, the clock ticks. Figure 3 shows how a light clock appears to an observer if both the clock and the observer are at rest.

Since the light signal traces out a path with the length of 2*L*, the period of the clock is given by

$$\begin{aligned} t=\frac{2L}{c}. \end{aligned}$$

**Fig. 4:**
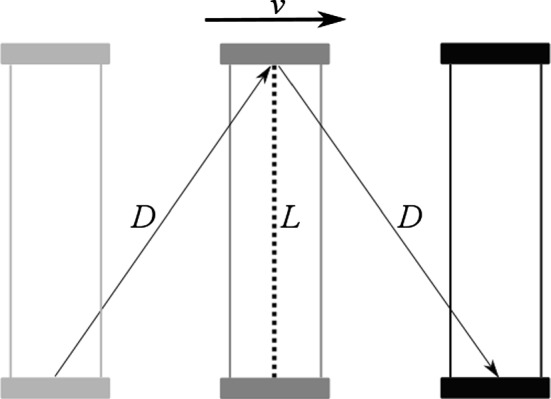
A light clock that is moving relative to an observer

But what happens if the light clock is set into rapid motion perpendicular to the rod? Now that the rod is moving, the light pulse must chase after the opposing end as it travels along the x-axis with velocity *v*. As can be readily seen from Fig. 4, this is to say that the light signal must cover a longer distance before it returns to its starting point. But since, according to the light principle, *c* is always constant, this also means that the light pulse needs more time to travel between the mirrors. As a consequence, the light clock ticks less often in the judgment of an observer who does not move with the clock. For an observer at rest, the period of the moving clock is given by

$$\begin{aligned} t^{\prime }=\frac{2D}{c}, \end{aligned}$$

where *D* is the hypotenuse to the perpendicular distance *L*. Using Pythagoras’ Theorem to calculate the exact length of *D*, we realize that the moving clock ticks at exactly half of the speed of a resting clock if $v=0.866c$. But even without doing any sums, the outcome of the TE should be apparent: A clock that is moving relative to an observer will be measured to tick slower than a clock at rest.

Of course, it might be tempting to assume that this result only affects timekeeping devices that rely on light. This, however, is not the case. On pain of violating the principle of relativity, *every* physical system that is a time-measuring device of sorts must slow down in keeping with the light clock. This is true not only of more traditional timekeeping mechanisms such as wound-up springs, swinging pendulums or oscillating crystals. Time dilation also affects the human metabolic system whose processes will slow down at precisely the same rate as the light clock. So, if we put a light clock and a living person on a spaceship traveling at 0.866*c*, then not only will the light clock be measured to tick at exactly half the speed of a resting lock. The person’s metabolism will slow to 50% of its normal rate as well, even though the slowing of all physical systems will be indiscernible from within the spaceship. However, if the person returns to earth after what we in the rest frame find to be one year, the person will have biologically aged only six months. Note also that the magnitude of this effect increases dramatically as velocities approach *c*: For instance, after an earth-year long voyage at 0.999*c*, a space traveller will have aged just a bit over 16 days.^11^

Let us return to the alternative universe of TFW. As I have pointed out, the frontline of the interstellar conflict can only be reached through collapsars, and in order to reach these, spaceships must cover several lightyears at velocities close to *c*. This, however, has serious repercussions for the soldiers who fight in this war. For instance, after Mandella returns from his first assignment after less than two years, he is handed out a table of organization (T/O) that almost pulls the rug from under his feet—and remember that his army career had already started in 1997:

I couldn’t get over the “20 Mar 2007” at the bottom of the T/O. I’d been in the army ten years, though it felt like less than two. Time dilation, of course: even with the collapsar jumps, traveling from star to star eats up the calendar. (Haldeman 1974, p. 70)

In light of what we have said about the implications of SR, it is not hard to figure out what happened. Mandella has spent the majority of his army career at velocities close to *c*.^12^ As a consequence, time has passed at a much slower rate in his reference frame, de-syncing him from the passage of time of his fellow humans on earth. While Mandella is only less than two years older after his first mission, ten full years have passed for the rest of humanity. In the beginning, he is not particularly concerned. For instance, since the remuneration of soldiers is calculated on the basis of earth-years, Mandella surmises that he will be a made man after his second mission:

After this [second] raid, I would probably be eligible for retirement with full pay—if I lived through the raid, and if they didn’t change the rules on us. A twenty-year veteran, and only twenty-five years old. (Haldeman 1974, p. 70)

But when Mandella returns to earth after a series of follow-up missions, he realizes that all the money in the world cannot compensate for the feeling of alienation he is experiencing. The year is 2458 and the war has been raging for almost half a millennium. The earth Mandella has left in the 1990s has not only evolved—it has ceased to exist. Social and political order have changed dramatically. For instance, in order to fight over-population, racial tensions and diseases, UNEF’s “Eugenics Council” has made heterosexual relationships and non-artificial reproduction a punishable offense (Haldeman 1974, p. 157). All babies are genetically engineered, brought up in government controlled crèches and trained to serve UNEF’s main goal, that of continuing a war that has already lost its military purpose, but without which earth’s economy would collapse (Haldeman 1974, p. 215). Quite generally, society has become a soulless dystopia in which people are treated as disposable commodities. On their seventieth birthday citizens receive a rating that is based on their estimated importance for society. If, like in the case of Mandella’s mother, the rating is zero, they are no longer eligible for any kind of medical care (Haldeman 1974, pp. 118–119).

Although Mandella has difficulty fitting into society, and even though most of his fellow humans view him with suspicion as well, his wartime heroics are heavily exploited by the UNEF-controlled media. Mandella—meanwhile by far the oldest surviving soldier and promoted to the rank of Major General—is expected to take command of his own strike force. But even army life leaves him with a feeling of isolation. Mandella is disliked by his subordinates for several reasons. His heterosexuality is considered an emotional dysfunction that is only tolerated because of his merits on the battlefield. And since he is hundreds of years older than everyone else in the common galactic frame, Mandella’s soldiers are required to learn twentieth-century English in order to be able to communicate with him. Mandella, who baffles his comrades with jokes about long-forgotten figures such as John Wayne (Haldeman 1974, p. 149), is as alien to his new lifeworld as Christopher Columbus or Hieronymus Bosch would be to ours.

For some time, Mandella has one last link to the world he knew. He is in a relationship with Marygay Potter, a female soldier who also entered the UNEF in the 1990s. But when the war is on a knife-edge, Mandella and Potter receive orders to embark on different missions in different parts of the galaxy. Never has SR been more gut-wrenching:

For a long time we couldn’t say anything. “I’m going to protest,” I said finally, weakly. [...] She was still struck dumb. This was not just a separation. Even if the war was over and we left for Earth only a few minutes apart, in different ships, the geometry of the collapsar jump would pile up years between us. When the second one arrived on Earth, his partner would probably be a half-century older; more probably dead. We sat there for some time, not touching the exquisite food, ignoring the beauty around us and beneath us, only conscious of each other and the two sheets of paper that separated us with a gulf as wide and real as death. We went back to Threshold. I protested but my arguments were shrugged off. I tried to get Marygay assigned to my company, as my exec. They said my personell had all been allotted. I pointed out that most of them probably hadn’t even been born yet. Nevertheless, allotted, they said. It would be almost a century, I said, before I even get to Stargate. They replied that Strike Force Command plans in terms of centuries. Not in terms of people. We had a day and a night together. The less said about that, the better. I wasn’t just losing a lover. Marygay and I were each other’s only link to real life, the Earth of the 1980s and 90s. Not the perverse grotesquerie we were supposedly fighting to preserve. When her shuttle took off it was like a casket rattling down into a grave. (Haldeman 1974, pp. 138–139)

## Physical and existential understanding

TEs facilitate understanding by requiring cognitive agents to construct mental representations of imaginary scenarios through which theoretical consequences can be made intelligible in an intuitive and non-technical manner. In order to achieve this aim, imaginary scenarios must be designed so as to strike a balance between familiarity and strangeness (Gooding 1992, p. 283): If, on the one hand, imaginary scenarios would not consist of familiar objects of everyday experience, cognitive agents would find it difficult to establish meaningful connections between theory and empirical fact. If, on the other hand, imaginary scenarios would not deviate from empirical reality through abstractions and idealizations, cognitive agents would fail to identify unforeseen theoretical consequences that in many cases remain hidden under normal lifeworld conditions. Einstein’s train TE is a case in point: We are familiar with objects such as wagons and train tracks. And we have no difficulty in constructing varying mental representations in which we perceive the unfolding scenario from two different viewpoints. Yet, if we introduce certain abstractions and idealizations into the imaginary scenario, we put ourselves into a position to make a far-reaching consequence of SR intuitively understandable.

On the view proposed here, much of what can be said about scientific TEs is also true of science fiction novels such as TFW. Like Einstein’s train scenario, the fictional world of TFW resembles ours in several important respects. For instance, we are familiar with the countless reports of the feelings of alienation and isolation that were common among returning Vietnam veterans. While soldiers from earlier conflicts were generally welcomed as heroes in what was seen as a necessary cause, many of those who had served in Vietnam returned to a society which had changed deeply during their absence. The US were greatly divided over the war, and the strong antiwar sentiment that could be felt all across the country was just one facet of a larger process of societal change (Tischler 2002). Apart from the physical and mental scars of war, it was this transformation of society that made it notoriously hard for Vietnam veterans to re-adapt into civilian life. Although it would be pretentious to think that we can recreate the emotional and social hardships of veterans without having experienced war and its aftermath ourselves, it seems safe to say that we can relate to the underlying feelings of isolation and alienation—feelings that are so central to human existence that we have no difficulty in identifying with them.

Another phenomenon that connects the fictional world of TFW with ours is the experience of parting ways with a loved one. We are familiar with the feelings of sadness and loss when we bid farewell at a train station or departures hall. And we can imagine how much stronger these feelings must be if the loved one not just goes on a business trip, but embarks on a mission that involves life-threatening risks. Like in the earlier example of returning Vietnam soldiers, we are dealing here with what could be called “existential feelings”. By “existential feeling”, I mean mental and emotional experiences that are so deeply embedded in human nature that they appear to be invariant across cultures and times. They are an essential part of the human condition, which is also why we can relate to them fairly easily.^13^

Upon reading TFW, we are invited to immerse ourselves in a fictional world that is sufficiently similar to ours. The main protagonist, from whose perspective the story is told, undergoes emotional experiences to which we can relate because of their existential character. However, it is through the introduction of several omissions^14^ and counterfactual deformations that the cognitive value of engaging with the fictional world of TFW is increased significantly. Instead of just offering a more or less realistic rendition of the emotional hardships of a returning soldier, TFW helps us to understand how existential feelings of alienation, isolation or loss would be amplified if technological innovations made relativistic effects like time dilation part of our every-day lifeworld. Even if we are fortunate enough to have never experienced war first-hand, we can relate to the former Vietnam soldier Larry Heinemann, who recalls that, after returning to the US, “I had the distinct feeling (common among returning veterans I think) that this was not my country, not my time” (Heinemann, quoted in Isaacs 1997, p. 12). However, after having immersed ourselves into the fictional world of TFW by putting ourselves into the shoes of William Mandella, we understand how much stronger this sentiment must be if the war zone was not on the other end of the globe, but lightyears away on the other end of the galaxy. While Heinemann’s statement might be seen as a slightly exaggerated figure of speech, it would be literally true if uttered by William Mandella.

Seen from this perspective, then, science fiction novels such as TFW indeed function much like TEs: In order to gain cognitive purchase from TFW, the reader must construct a mental representation of a fictional world which is designed to strike a balance between strangeness and familiarity. Once the reader has successfully immersed herself into this world by empathizing with the main protagonists, she can observe how the narrative unfolds according to its own inner logic. Upon doing this, the reader puts herself into a position to make certain unforeseeable consequences of SR intelligible: The reader comes to understand what it would mean for human beings to inhabit a world in which relativistic effects like time dilation directly interfere with their everyday lives.^15^

It is now possible to address an issue to which I have already alluded in the opening section. On the view proposed here, TEs in physics aim at what I have called “physical understanding”. Following the results of Sect. 2, the process of facilitating physical understanding involves two key aspects: The first is to construct a mental representation of an imaginary scenario onto which certain theoretical concepts can be mapped. It is in this way that we use TEs to fix the physical meaning of concepts by specifying their referents. Once the theoretical concepts are brought under the agent’s cognitive control, the second stage consists of applying the newly acquired framework to the representation of the imaginary scenario, and record the outcome. Here, the point is to make certain consequences of the theory intuitively accessible within the confined space of the laboratory of the mind. Given this two-tiered structure, it is clear that the scope of scientific TEs is usually focused to what one could call “inner-theoretical clarification”: The purpose of most scientific TEs is to increase our understanding of the *inner workings* of theories as well as their immediate empirical consequences.^16^ TEs achieve this aim by establishing meaningful connections between abstract concepts and empirical fact, and by exemplifying the consequences of applying the theories to their intended target domains (cf. Stuart 2017).

Yet, looking at science fiction novels, a slightly different picture emerges. To be sure, if we wish to gain cognitive purchase from a novel like TFW, we are also required to actively participate in a distinctively designed imaginary scenario, for instance, by imagining to see the world of TFW through William Mandella’s eyes. However, the point of this exercise is neither to consolidate the physical meaning of abstract concepts, nor to exemplify certain theoretical consequences. Throughout TFW, Haldeman simply stipulates what the ramifications of time dilation are—beyond that, the narrative does nothing to promote physical understanding of why time dilation comes about. This might seem to undermine the analogy between science fiction and TEs at first. But it is important to realize that TFW *does* promote our understanding of SR, albeit in a way different from Einstein’s train or the light-clock TE. Instead of clarifying the inner workings and immediate empirical consequences of SR, TFW makes intelligible how certain implications of SR would affect the *emotional and social dimensions of human existence*.^17^ Hence, while scientific TEs such as Einstein’s train focus on inner-theoretical clarification and physical understanding, science fiction novels like TFW aim at promoting the *existential understanding* of SR. If written in a scientifically responsible manner,^18^ science fiction has thus the potential to increase our understanding of what it means for us, as human beings, to live in the kind of world our increasingly arcane scientific theories purport to describe.

There is one final point I would like to address. In Sect. 2, I have introduced a distinction between mathematical and physical understanding. Following the idea that the performability of different epistemic practices result in different kinds of understanding, it seems reasonable to assume that existential understanding is the result of a distinctive epistemic practice as well. Hence, the question arises: what can we say about the mental operations that must be performed in order for existential understanding to occur, and how are they different from those that facilitate physical understanding?

When a cognitive agent performs a scientific TE, she is required to construct a mental representation of a scenario onto which certain theoretical concepts can be mapped, and through which certain theoretical consequences can be exemplified. In many cases—like Einstein’s train or the light-clock TE—it is also necessary to imagine an observer from whose perspective the outcome of the TE is perceived. On closer inspection, however, the role of the observer turns out to be confined to that of a mere data collector. For instance, the outcome of Einstein’s train TE does not depend on whether the cognitive agent imagines the observers to be herself, other conscious beings or measurement apparatuses. As long as the temporal order of the incoming light flashes is correctly recorded, the specifics of the imagined observer are entirely irrelevant for the purpose of facilitating physical understanding.

Yet if we look at science fiction novels such as TFW, things stand differently. To be sure, in order to gain cognitive purchase from TFW, we are also required to construct a mental representation of a fictional world that is designed to strike a balance between strangeness and familiarity. But if all we were to imagine was a world that is technologically superior to ours, this would lead us nowhere as far as existential understanding is concerned. The reason for this is straightforward: If my analysis is correct and if the aim of TFW is to make intelligible how certain consequences of SR would affect the emotional and social dimensions of *human existence*, then, of course, the fictional world of TFW must be imagined so as to contain beings who are able to undergo experiences that are relatable due to their existential character. For instance, not much could be learned from TFW if we replaced William Mandella with Lieutenant Commander Data from *Star Trek: The Next Generation*.^19^ And this tells us something significant about the mental operations that must be performed in order to gain cognitive purchase from TFW: In order for existential understanding to occur, we are required to construct a mental representation of William Mandella in his environment. In part, this means to imagine a technologically advanced world in which interstellar travel is a reality. Yet, even more important, it also means to construct a representation of Mandella’s system of thoughts, beliefs, desires and feelings, and to identify with this representation through what Goldman has called “enactment imagination” (Goldman 1998). This is to say that we are required to use the faculties of imagination to recreate the phenomenology of particular mental states by producing facsimiles of the selected states in our own minds. So, for instance, when we consider the scene in which Mandella watches the takeoff of his spouse’s shuttle, we are required to take up his perspective by enacting or re-creating feelings of loss and sadness, feelings to which we can relate due to their existential character. The cognitive surplus that can be gained from TFW is achieved by way of manipulating the mental representation: The process of enactment imagination that makes the identification with the main character possible is driven by background knowledge about the specific conditions under which Mandella watches his partner’s departure. Since we know that time dilation will most likely turn their farewell into a final parting, we come to understand how certain consequences of SR would affect the human lifeworld by amplifying existential feelings such as loss, sadness or isolation.

Let me conclude this section with a summary. On the view defended here, the kind of physical understanding that is brought about by TEs and the kind of existential understanding that is facilitated by science fiction novels are structurally similar in several important respects. To begin with, TEs and science fiction novels are similar in that neither pay epistemic dividends if they are merely passively consumed. In order to yield understanding, both require their recipients to perform operations whose aim it is to construct mental representations that are specifically designed to strike a balance between strangeness and familiarity. The productive interplay between strangeness and familiarity is another common trait that is instrumental to the epistemic outcome in both areas: If the respective mental presentations are too strange, then cognitive agents will find it hard to establish meaningful connections between the already familiar and that which is still beyond their cognitive grasp. If, on the other hand, the respective mental representations are too familiar, then they will fail to disclose anything of epistemic interest. Hence, it is only if “there is enough strangeness to disturb and enough familiarity to be accessible” (Gooding 1992, p. 283) that both TEs and science fiction novels can be successful in exemplifying the consequences of abstract scientific theories in an intuitive, quasi-sensory and non-technical manner. Once intelligibility is achieved in this way, cognitive agents are also in a position to deepen their understanding by further manipulating the respective mental representation. In the case of TEs, cognitive agents could, for instance, proceed by manipulating the imaginary scenario so as to produce variations with decreasing degrees of specificity. It is in this way, as we have seen, that TEs can help to make the physical meaning of mathematical concepts intelligible. In the case of science fiction novels, cognitive agents could proceed by manipulating the initial mental representation so as to produce “mixed narratives”, i.e. narratives that combine fictional elements with actual memories and experiences. For instance, upon immersing myself into the parallel universe of TFW, I could reflect on how my family life would have been affected if my last research stay had not led me to another continent, but to a solar system thousands of light years away from Earth.

Despite all similarities, however, one must not lose sight of the differences between the kinds of understanding that are facilitated by TEs and science fiction novels. In order to fix the meaning of abstract concepts, many TEs require us to construct imaginary scenarios in which familiar objects of every-day experience interact under hypothetical or counterfactual conditions. Once the imaginary scenario is set up in the expected manner, cognitive agents can then let the scenario unfold in order to make the consequences of applying the newly acquired theoretical framework intelligible. At first glance, this way of proceeding does not appear to be significantly different from what happens when we gain cognitive purchase from a science fiction novel such as TFW: Here too, we are required to construct a mental representation of a fictional world in which hypothetical or counterfactual conditions hold, and through which certain consequences of SR become intuitively intelligible. However, while the focus of TEs is on familiar quasi-empirical objects and the conceptual frameworks that are being used to describe them, science fiction novels like TFW are primarily concerned with human beings who undergo familiar existential feelings while being exposed to an environment in which the consequences of SR are directly experienceable. This difference in focus is reflected in the different kinds of operations that underlie physical and existential understanding: In order to obtain the latter, cognitive agents must empathize with William Mandella by re-creating familiar feelings of loss, alienation or sadness. If the mental representation of Mandella’s system of thoughts, beliefs, desires and feelings is integrated into the parallel universe of TFW, we come to understand how certain consequences of SR would affect the social and emotional dimensions of human existence.

## Concluding remarks

Edmund Husserl is famous for the claim that modern scientific culture is haunted by a deeply-rooted crisis (Husserl 1970). Despite all their sophistication and effectiveness, the special sciences—and mathematical physics in particular—have become increasingly remote from human experience. As a consequence, non-scientists find it more and more difficult to understand what modern theories tell us about the world of every-day practice. And, according to Husserl, this results in a growing skepticism about the principal worth of the scientific enterprise and thus in a menacing irrationalism: “In our vital need—so we are told—this science has nothing to say to us.” (Husserl 1970, p. 6)

Husserl—a German philosopher of Jewish descent—wrote these lines during the 1930s and hence during a time when the forces of nationalism, fanaticism and anti-scientific obscurantism already threw a dark shadow over Europe. Yet, in light of the current rhetorics of “alternative facts” and “post-truth politics”, it is hard to not feel addressed by Husserl’s message. The authority of science is again coming under fire and it is reasonable to suspect that ignorance about what science is and says is part of the problem. Forbidding mathematical formalisms, highly complex technological apparatuses and inaccessible technical jargons are among the hurdles that prevent laypersons not only from grasping the content of particular scientific theories; the increasingly common trend to reject, say, climate science because “it is only a model” attests to an even more fundamental ignorance about the very nature of scientific reasoning. Now, although I am not suggesting that TEs and science fiction alone will be able to save the day, I do believe that they could go some way toward closing the gap between the general public and professional scientists. As we have seen, TEs are highly efficient tools for conveying scientific ideas to non-experts. Since, in most cases, little pre-existing knowledge is necessary for their execution, and since most TE scenarios consist of familiar objects of every-day experience, TEs prove epistemically rewarding to a wide array of cognitive agents. What is more, since TEs must be actively done and not just passively consumed, TEs afford a sense of accomplishment that motivates cognitive agents to cultivate their critical thinking and analytical skills. Given all this, it is not surprising that TEs have been praised for their positive impact on science education (cf., e.g., Gilbert and Reiner 2010).

However, there is an important sense in which the epistemic reward facilitated by TE is only partial. If my analysis is correct, then one of the primary aims of many TEs is to consolidate the meaning of abstract concepts by requiring the cognitive agent to perform operations through which these concepts are mapped onto mental representations of scenarios that consist of familiar objects of our every-day experience. Yet, even though they are built up from familiar objects, most TE scenarios are still very far removed from the world in which we, as human beings, live our daily lives. As water buckets in empty universes and cars at close-to-light-speed velocities attest, much of the epistemic force of TEs stems from the fact that familiar objects are imagined under conditions that, more often than not, differ radically from those prevailing in the lifeworld of every-day practice. Now, to be sure, my point is not that the strangeness of most TE scenarios is a reason for concern. Quite the opposite, the deliberate distortion of reality is, as I have argued, crucial for the epistemic outcome of TEs. But the drawback of invoking imaginary scenarios in which fairly strange conditions hold is that cognitive agents might find it hard to see how anything that happens under such conditions has practical relevance for the proverbial “man on the street”. To put it bluntly: It may be an amusing pastime to fix the meaning of abstract concepts by pondering over cars that move with velocities close to *c*. But what difference do these concepts make for someone who is stuck in morning traffic six times a week?

While TEs are a highly effective tool to make scientific theories intelligible, they do little to counterbalance the increasingly common sentiment that much of today’s science is too detached from the real world to make a tangible impact on problems real people face. In my view, it is precisely at this juncture that science fiction could come to the rescue. To be sure, science fiction novels also take place in strange fictional worlds that differ from ours in more or less drastic ways. However, since novels such as TFW aim at existential rather than at physical understanding, their epistemic utility does not primarily depend on imagining strange scenarios, but on constructing mental representations of desires and feelings real people could have, and to which we can relate due to their existential character. William Mandella is imagined as such a real person who experiences emotions that virtually everyone of us has experienced at some point in life. Using the imagination to put Mandella into a specifically designed environment not only allows us to understand how certain consequences of scientific theories would transform our lifeworld by amplifying familiar feelings such as loss or alienation. By empathizing with a real person who acts under conditions that, although not the ones we are used to, are specified by our best scientific theories, we also come to understand that the seemingly arcane “world of science” might only be a few technological innovations away from becoming the world in which our daily lives unfold. Seen from this perspective, then, TEs and science fiction indeed appear to be natural bedfellows: While the former are highly effective tools for facilitating physical understanding of theories and/or the phenomena in their domain, the capacity of science fiction to generate existential understanding could help to give science a more human face.

## Footnotes

1 This line of argument can also be found in the debate about TEs. For instance, Jim Brown writes that “explanation [is] an objective feature of theories [whereas] [u]nderstanding is more subjective, a psychological feature of ourselves” (Brown 2014, p. 372).

2 Of course, much depends on what one takes understanding to be. For instance, if one subscribes to the view that understanding consists in the “grasping of relations between items of information” (Kvanvig 2003, p. 197), the cognitive outcome of my reading of Orwell’s novel *is* the acquisition of new knowledge, namely knowledge about the relations between existing items of factual information about the world. The account of understanding that I am advocating will be outlined in the next section.

3 Note, however, that Hills’ account is more narrow than the manipulationist approach I am advocating here. While Hills agrees that understanding is a certain kind of ability or know-how, she is only concerned with the intellectual abilities that are necessary to give explanations of why *P*.

4 Of course, the assumption here is that the mental representation captures the relevant features of what is being represented. If this is the case, the ability to manipulate the mental representation of *P* prefigures the possibility of successfully manipulating *P*. It should also be noted that the notion of manipulation that is at work here must be sufficiently inclusive to incorporate a broad range of disparate objects. If the object of understanding is, say, an engine, “manipulation” will amount a re-arrangement, replacement or patching of its functional parts. If the object of understanding is, say, a mathematical proof, “manipulation” will predominantly consist in drawing inferences. As we shall see in more detail later, “manipulation” in the case of TEs usually amounts to the construction and re-arrangement of imaginary scenarios and to the ability to draw meaningful inferences from them.

5 As one reviewer has noted, it might not be immediately clear why the the ability to construct a mental representation solves the problem of counterfeit understanding. Let me illustrate the basic idea by means of an example: Suppose you want to test student A’s and B’s understanding of elementary algebra by asking them to give a step-by-step solution of the equation $2x+1=3$. But imagine furthermore that, since one of your test sheets have miraculously disappeared, you have reason to believe that one of your students already knew the question prior to the exam. During the exam, both students perform the same operations: after moving the “$+\,1$” from left to right, making it a “− 1”, they move the “2” from left to right, making it a “$\frac{1}{2}$”, and finally obtain the solution $x=1$ by simplifying the right side. Yet, even though both students have displayed the exact same ability to perform according to set standards, you want to make sure that neither of them has merely simulated understanding by replicating a pre-memorized solution. Several strategies suggest themselves: you could, for instance, inquire as to why the “2” from step 2 becomes a “$\frac{1}{2}$” and not a “$-\,2$”; or you could ask your students to solve a different, but sufficiently similar equation like $3x+5x+4-x+7=88$. But why is it that such follow-up questions are an appropriate way to distinguish counterfeit from genuine understanding? In my view, and in line with Wilkenfeld’s views on the matter, the most plausible answer is that these follow-up questions are designed to look for cognitive structures that prefigure worldly abilities like solving equations on a blackboard. The most promising candidate for such cognitive structures are mental representations of the object one seeks to understand, in this case a mental representation of the relevant parts of elementary algebra with which one can do certain things, and which must stand in the right relations with other mental representations like that of the relevant parts of arithmetic. The prediction that follows from this hypothesis is that a student who only replicates pre-memorized operations will not be able to answer the follow-up questions in a satisfactory manner. A student who is able to construct a mental representation of the relevant parts of algebra, on the other hand, will not only be able to solve the initial equation. She could also solve indefinitely many other algebraic equations, contemplate alternative solution-strategies before actually writing one of them down, talk about elementary algebra in her own words, or perhaps even—if the mental representation of algebra is sufficiently well integrated into her global representation—apply algebra to empirical situations such as weights representing “$2x+1$” and “3” on each pan of a balancing scale. Cf., for empirical studies detailing how different areas of mathematics are mentally represented in the human brain, e.g. Greeno (1983), Dehaene (1999), and Dreyfus (2002).

6 While I believe that manipulationism is the most promising approach, it is beyond the scope of this paper to defend it against possible alternatives. For criticisms of manipulationist accounts of understanding, cf., e.g., Khalifa (2017, chapter 3).

7 I consider this to be a well-established historical fact. For instance, in his historical account of the global dispersion of Feynman diagrams in post-war physics, David Kaiser describes how their reception in the Soviet Union was hindered by the cold war separation of Western and Soviet physicists. Although Soviet theorists had access to published papers, travel bans “deprived them of any chance for informal, face-to-face exchanges with physicists on the other side of the iron curtain”. This lack of personal contact made it impossible for them “to pick up and use Feynman diagrams in anything like the ways their colleagues in the United States, Great Britain, and Japan did” (Kaiser 2005, p. 150).

8 As one reviewer has pointed out, there are exceptions to this generalization, most notably the famous Einstein–Podolsky–Rosen TE that was designed to show that quantum theory is incomplete. Although I agree that familiar objects of every-day experience do not seem to play a decisive role in this TE, I am wondering whether this tells us more about quantum mechanics than about TEs. After all, it is remarkable that the Einstein–Podolsky–Rosen TE is one of the few TEs in quantum mechanics in which quantum phenomena are not modeled onto every-day scenarios (like, for instance, in Schrödinger’s cat), but in which the quantum phenomena themselves are the objects of TE reasoning. However, since discussing this issue would lead me too far afield, all I can do here is to refer the reader to more detailed studies such as, e.g., McAllister (1996, pp. 248–250), Van Dyck (2003) or Peacock (2018).

9 What I have in mind here is this: In part, the didactic value of TEs stems from the fact that they provide us with exemplary scenarios in which the application of abstract concepts becomes experienceable in an intuitive, quasi-sensory and oftentimes first-personal manner. After having imagined myself in two different places within the same imaginary scenario, the outcome of the train TE becomes directly observable without the need for any kind of higher-order cognitive skills such as mathematical abilities. But, of course, it is possible to achieve similar results by relying on imaginary scenarios with higher degrees of abstractness. For example, trains, wave-like light flashes and embodied observers could be replaced by rigid rods, ideal clocks and detection devices. These could then be replaced by systems of coordinates, which in turn could be replaced by equations that govern the transformations between frames quantitatively. On my view, such an “ascent from embodied sensation” (Gooding 1992, p. 294) to increasingly abstract systems of representation is a very effective way to make the physical meaning of mathematical concepts and techniques intelligible (cf., also, Giere 1988; Nersessian 2005).

10 One reviewer has wondered whether the kind of manipulationism I am advocating would be improved by replacing all references to mental representations with concepts, thus reducing “*S* *understands P*” to “*S* *possesses the concepts needed to make relevant inferences about P*”. Although much more would have to be said about this complex issue (cf., e.g., Machery 2009; Margolis and Laurence 2011), my response is twofold: Whether a manipulationist account would benefit from replacing mental representations with concepts depends, of course, on what one takes concepts to be. While there is little agreement among philosophers, it seems very common among psychologists to “refer to a concept as a mental representation that is used to meet a variety of cognitive functions” (Solomon et al. 1999, p. 100). Following psychologists on this not only seems to be the scientifically less controversial move. It also makes mental representation the more general term, allowing for the possibility that we sometimes understand by means of one kind of mental representation (concepts) and sometimes by means of others (e.g. imaginary scenarios). Concerning the issue of concept possession, I agree that a good account of understanding should also be an account of concept possession, and that one of the primary aims of TEs is to bring concepts under one’s cognitive control. However, and this is particularly important in philosophy of science, a distinction must be made between *merely* and *genuinely* possessing a concept. While knowing textbook definitions might be sufficient for the former, genuine possession of a concept like “reference frame” requires the ability to use it. Hence, although concept possession is important, manipulation is, in my view, more fundamental.

11 The phenomenon of time dilation seems to give rise to several paradoxical consequences, the “twin paradox” being the most famous one. Imagine two identical twins, one of whom travels through space at 0.999*c* for one earth-year, while the other stays on earth. Since, according to the principle of relativity, there is no privileged frame of reference, each twin could be described to move away from the other at a velocity close to *c*. If this were the case, this would lead to the paradoxical consequence that, upon their reunion, each twin should find the other to have aged significantly less. However, the problem with this way of looking at things is that the spacetime paths of the two twins are in fact asymmetrical. Since the traveling twin must turn around in order to come back to earth, her trajectory involves at least two inertial frames and several accelerations and decelerations. Cf., for more details, e.g. Luminet (2010).

12 It should be added that the time dilation effects are further magnified by the fact that soldiers spend much of their traveling time in cryogenic deepfreeze.

13 As one reviewer has pointed out, notions such as “existential feeling” or “existential understanding” could be taken to allude to the works of thinkers that are commonly associated with Existentialism. Even though I agree that it would be worthwhile to contrast my manipulationist account with, for instance, Heidegger’s conception of understanding (cf., e.g. Wrathall 2013), or to expand my discussion of existential understanding by Heidegger’s or Merleau-Ponty’s “existential conception of science” (cf., e.g., Rouse 1986), doing so would require much more space than I have here.

14 Haldeman ignores several factors that currently stand in the way of making interstellar travel a reality. Besides the fact that the available methods of spaceship propulsion do not provide nearly enough kinetic energy to reach velocities close to *c*, factors such as the presence of high-energy, ionizing cosmic ray nuclei present serious challenges for ensuring the health of human space travelers (cf., e.g., Setlow 2003).

15 This, of course, is not to say that relativistic effects aren’t already interfering with our lifeworld. For instance, since clocks on satellites are affected by their high speeds, global navigation systems would not deliver sufficiently accurate results if time dilation was ignored.

16 Even tough I believe that inner-theoretical clarification is the most common achievement of TEs in science, one might, for example, point to Stevin’s chain as a TE that *produces* rather than clarifies a theory.

17 One could wonder whether it is really possible to understand how SR affects the emotional and social dimensions of human existence without also understanding how time dilation comes about. On my view, this is indeed possible: In order to gain existential understanding of SR, a cognitive agent only needs to have *factual knowledge* that time dilation exists, that it occurs at speeds close to *c* and that it interferes with the aging processes of subjects in different frames. Although physical understanding of time dilation might be a bonus, it is not necessary in order to understand how existential feelings such as alienation would be amplified by being de-synced from the rest of humanity.

18 Science fiction that is rigorous in its use of scientific theories is commonly referred to as “hard” science fiction. It should be apparent that my argument only applies to this sub-genre and not to more fantastic variants of science fiction.

19 Data is a human-like, self-conscious android with exceptional computational capabilities, but without the ability to experience emotions of any kind.

## References

[CR1] Brown JR (2014). Explaining, seeing, and understanding in thought experiments. Perspectives on Science.

[CR2] Brown, J. R., & Fehige, Y. (2017). Thought experiments. In E. N. Zalta (Ed.), *Stanford Encyclopedia of Philosophy*. Summer 2017. https://plato.stanford.edu/archives/sum2017/entries/thought-experiment/. Accessed 13 July 2019.

[CR3] Carroll N (2002). The wheel of virtue: Art, literature, and moral knowledge. Journal of Aesthetics and Art Criticism.

[CR4] Chang H, De Regt H, Leonelli S, Eigner K (2009). Ontological principles and the intelligibility of epistemic activities. Scientific understanding: Philosophical perspectives.

[CR5] Chang H (2011). The philosophical grammar of scientific practice. International Studies in the Philosophy of Science.

[CR6] Davenport EA (1983). Literature as thought experiment (on aiding and abetting the muse). Philosophy and the Social Sciences.

[CR7] Davies D (2007). Thought experiments and fictional narratives. Croatian Journal for Philosophy.

[CR8] De Regt HW (2017). Understanding scientific understanding.

[CR9] De Regt HW, Dieks D (2005). A contextual approach to scientific understanding. Synthese.

[CR10] De Regt HW, Leonelli S, Eigner K (2005). Scientific understanding: Philosophical perspectives.

[CR11] De Smedt J, De Cruz H, French PA, Wettstein HK (2015). The epistemic value of speculative fiction. Midwest studies in philosophy.

[CR12] Dehaene S (1999). Sources of mathematical thinking: Behavioral and brain-imagining evidence. Science.

[CR13] Dreyfus T, Tall D (2002). Advanced mathematical thinking processes. Advanced mathematical thinking.

[CR14] Egan D (2016). Literature and thought experiments. Journal of Aesthetics and Art Criticism.

[CR15] Einstein A (2005). Relativity. The special and general theory.

[CR16] Elgin CZ, Gibson J, Huemer W, Pocci L (2007). The laboratory of the mind. A sense of the world: Essays on fiction, narrative, and knowledge.

[CR17] Elgin CZ (2014). Fiction as thought experiment. Perspectives on Science.

[CR18] Ellis GFR, Williams RM (1998). Flat and curves space times.

[CR19] Faraoni V (2013). Special relativity.

[CR20] Giere R (1988). Explaining science: A cognitive approach.

[CR21] Gilbert JK, Reiner M (2010). Thought experiments in science education: Potential and current realization. International Journal for Science Education.

[CR22] Goldman A (1998). Simulating minds: The philosophy, psychology and neuroscience of mindreading.

[CR23] Gooding DC, Hull D, Forbes M, Okruhlik K (1992). What is experimental about thought experiments?. Proceedings of the biennial meeting of the Philosophy of Science Association.

[CR24] Gordon J (2006). Joe Haldeman.

[CR25] Greeno JG, Gentner D, Stevens AL (1983). Conceptual entities. Mental models.

[CR26] Grimm SR, Baumberger C, Ammon S (2017). Explaining understanding. New perspectives from epistemology and philosophy of science.

[CR27] Haldeman J (1974). The Forever War.

[CR28] Hills A (2016). Understanding why. Nous.

[CR29] Husserl E (1970). The crisis of the European sciences and transcendental phenomenology. An introduction to phenomenological philosophy.

[CR30] Isaacs AR (1997). Vietnam shadows. The war, its ghost, and its legacy.

[CR31] Kaiser D (2005). Drawing theories apart. The dispersion of Feynman diagrams in postwar physics.

[CR32] Khalifa K (2017). Understanding, explanation, and scientific knowledge.

[CR33] Kuhn TS (1970). The structure of scientific revolutions.

[CR34] Kvanvig J (2003). The value of knowledge and the pursuit of understanding.

[CR35] Luminet J-P, Callender C (2010). Time, topology, and the twin paradox. The Oxford handbook of philosophy of time.

[CR36] Machery E (2009). Doing without concepts.

[CR37] Margolis, E., & Laurence, S. (2011). Concepts. In E. N. Zalta (Eds.), *Stanford Encyclopedia of Philosophy*. Summer 2017. https://plato.stanford.edu/archives/sum2017/entries/concepts/. Accessed 13 July 2019.

[CR38] McAllister JW (1996). The evidential significance of thought experiments in science. Studies in History and Philosophy of Science.

[CR39] Nersessian NJ, Jones MR, Cartwright N (2005). Abstraction via generic modeling in concept formation in science. Idealization XII: Correcting the model. Idealization and abstraction in the sciences.

[CR40] Peacock KA, Brown JR, Fehige Y, Stuart MT (2018). Happiest thoughts. Great thought experiments of modern physics. The Routledge companion to thought experiments.

[CR41] Polanyi M (1998). Personal knowledge. Towards a post-critical philosophy.

[CR42] Rouse J (1986). Merleau-Ponty and the existential conception of science. Synthese.

[CR43] Setlow RB (2003). The hazards of space travel. EMBO Reports.

[CR44] Solomon KO, Medin DL, Lynch E (1999). Concepts do more than categorize. Trends in Cognitive Sciences.

[CR45] Sorensen R (1992). Thought experiments.

[CR46] Stuart MT (2016). Taming theory with thought experiments: Understanding and scientific progress. Studies in History and Philosophy of Science.

[CR47] Stuart MT (2017). Imagination: A sine qua non of science. Croatian Journal for Philosophy.

[CR48] Stuart MT, Brown JR, Fehige Y, Stuart MT (2018). How thought experiments increase understanding. The Routledge companion to thought experiments.

[CR49] Swirski P (2007). Of literature and knowledge: Explorations in narrative thought experiments, evolution and game theory.

[CR50] Tischler B, Young MB, Buzzanco R (2002). The antiwar movement. A companion to the Vietnam war.

[CR51] Van Dyck M (2003). The roles of one thought experiment in interpreting quantum mechanics. Werner Heisenberg meets Thomas Kuhn. Philosophica.

[CR52] Wilkenfeld DA (2013). Understanding as representation manipulability. Synthese.

[CR53] Wrathall M, Wrathall M (2013). Heidegger on human understanding. The Cambridge companion to being and time.

